# Lower limb joint loading in patients with unilateral hip osteoarthritis during bipedal stance and the effect of total hip replacement

**DOI:** 10.3389/fbioe.2023.1190712

**Published:** 2023-06-16

**Authors:** S. van Drongelen, J. Holder, F. Stief

**Affiliations:** ^1^ Department of Orthopedics (Friedrichsheim), University Hospital Frankfurt, Goethe University Frankfurt, Frankfurt, Germany; ^2^ Dr. Rolf M. Schwiete Research Unit for Osteoarthritis, Department of Orthopedics (Friedrichsheim), University Hospital Frankfurt, Goethe University Frankfurt, Frankfurt, Germany

**Keywords:** posture, ground reaction forces, external hip adduction moment, external knee adduction moment, symmetry angle

## Abstract

Osteoarthritis of the hip is a common condition that affects older adults. Total hip replacement is the end-stage treatment to relief pain and improve joint function. Little is known about the mechanical load distribution during the activity of bipedal stance, which is an important daily activity for older adults who need to rest more frequently. This study investigated the distribution of the hip and knee joint moments during bipedal stance in patients with unilateral hip osteoarthritis and how the distribution changed 1 year after total hip replacement. Kinematic and kinetic data from bipedal stance were recorded. External hip and knee adduction moments were calculated and load distribution over both limbs was calculated using the symmetry angle. Preoperatively, the non-affected limb carried 10% more body weight than the affected limb when standing on two legs. Moreover, the mean external hip and knee adduction moments of the non-affected limb were increased compared to the affected limb. At follow-up no significant differences were observed between the patients’ limbs. Preoperative and postoperative changes in hip adduction moment were mainly explained by the combination of the vertical ground reaction force and the hip adduction angle. Stance width also explained changes in the hip and knee adduction moments of the affected leg. Furthermore, as with walking, bipedal standing also showed an asymmetric mechanical load distribution in patients with unilateral hip osteoarthritis. Overall, the findings suggest the need for preventive therapy concepts that focus not only on walking but also on optimizing stance towards a balanced load distribution of both legs.

## 1 Introduction

Osteoarthritis (OA) is a leading cause of disability and imposes societal costs in older adults (Hunter and Bierma-Zeinstra, 2019). It is even more prevalent than in previous decades due to an ageing and increasingly obese population. Primary total hip replacement (THR) is the standard treatment for end-stage hip OA, providing pain relief and improved joint function. The demand and volume of this procedure is predicted to increase in the coming years due to higher demand for improved mobility and quality of life in the aging population (Maradit Kremers et al., 2015).

In patients with unilateral hip OA, a pain-induced protective walking pattern results in uneven loading of the lower extremities. Pathological moments in the hip and knee joint during walking have been noted (Hurwitz et al., 1997; Shakoor et al., 2011; Schmidt et al., 2017). Despite good clinical-functional outcomes (Neuprez et al., 2020), some studies found that gait kinematics (i.e., reduced hip extension) and kinetics (i.e., lower knee adduction moments) did not normalize after THR (Foucher et al., 2007; Stief et al., 2018). The development of OA in the hip joint has been shown to be related to increased joint loading during walking (Hurwitz et al., 2001). In this context, the external hip adduction moment (HAM) has been identified as one of the most important determinants of hip contact force and joint loading (Lenaerts et al., 2009; Wesseling et al., 2015). Peak external knee adduction moments (KAM) are associated with the rate of progression and severity of knee OA (Sharma et al., 1998; Miyazaki et al., 2002). Patients with hip OA have also been shown to have altered lower limb joint mechanics during sit-to-stand tasks (Eitzen et al., 2014; Abujaber et al., 2015) and stair climbing (Queen et al., 2015), suggesting that these activities may contribute to the development of OA in adjacent joints (Jungmann et al., 2015; Joseph et al., 2016).

Although standing is an important activity of daily living (Morlock et al., 2001), there is limited information on lower extremity mechanical load distribution in patients with unilateral hip OA. It appears that patients with unilateral hip OA shift more weight to the non-affected leg during standing (Talis et al., 2008; Miura et al., 2018). However, in these studies, leg loading is expressed only as asymmetry between the legs or as uneven distribution of vertical ground reaction force between the legs. There is no information on the hip or knee joint moments of the affected and non-affected leg, nor is there detailed information on what factors influence asymmetrical leg loading. When walking on flat surfaces, patients with unilateral hip OA use compensatory strategies (i.e., greater foot progression angle and increased lateral trunk displacement toward the affected side) that directly affect HAM and KAM (Schmidt et al., 2017). It is therefore reasonable to assume that some of these compensatory strategies also occur during bipedal standing. If this is the case, the symmetry of lower limb joint moments may also be affected. Characteristic gait changes to unload the affected limb include increased lateral trunk displacement (LTD) toward the affected side (Reininga et al., 2012), altered foot progression angle (FPA) at the affected limb (Müller et al., 2012) and increased stride width (Stief et al., 2021). Information on these compensatory strategies for bipedal standing is missing, although their effects on lower limb joint moments may have implications for rehabilitation of patients with hip OA. Rehabilitation focusing on motor control to move and stand more symmetrically could be applied to patients with hip OA and after THR to modify motor strategies (Boonstra et al., 2011). This is in line with Hunter and Bierma-Zeinstra (2019), who stated that management of OA should shift from reactive to proactive and preventive measures.

The aim of the present study was to investigate load distribution before THR and the improvement in load distribution after THR during bipedal standing in patients with unilateral hip OA and finally to examine whether kinematic and kinetic variables in general and compensatory strategies in particular correlate with significant changes in joint loading. It was expected that before THR, the non-affected limb would experience greater lower limb joint moments than the affected limb in patients with unilateral hip OA. Furthermore, it was hypothesized that joint loading asymmetries in patients with unilateral hip OA would differ from those in a healthy control (HC) group and would be due in part to changes in kinematic and kinetic variables.

## 2 Methods

In the present study, data from patients who had participated in previous prospective studies in our clinic were analyzed (Schmidt et al., 2017; Stief et al., 2018; van Drongelen et al., 2019; van Drongelen et al., 2020). In these studies, gait analysis and radiography were performed preoperatively and 1 year after THR. The complete protocol for these studies has been described previously (Stief et al., 2018; van Drongelen et al., 2019). In addition, data from HCs with a similar age distribution that were available in our database were used for comparison.

### 2.1 Participants

Symptomatic patients with radiologically confirmed unilateral hip OA (Kellgren-Lawrence > 2) between the age of 30 and 80 years, who were scheduled for and received THR were considered for inclusion. Exclusion criteria were: OA of lower limb joints other than the affected hip, chronic or neuromuscular diseases, history of orthopedic surgery of the lower extremities, and use of assistance devices during walking. Only data from patients who had three valid trials of two-leg-standing measured during gait analysis in the week before and 1 year after surgery were included. Patients with a body mass index (BMI) > 35 kgm^−2^ were excluded from the analyses. Finally, data from 43 patients were included in the study (Table 1).

**TABLE 1:** Anthropometric data, kinetic data and kinematic data of patients with unilateral hip osteoarthritis and healthy controls.

	Healthy controls	Hip OA patients preoperatively		Hip OA patients postoperatively	
Anthropometrics					
Number of participants	17	43		43	
Males/Females	8/9	24/19		24/19	
Age (years)	56.0 (52.5–67.0)	63.0 (53.0–69.0)†		64.0 (54.0–70.0)	
Body mass (kg)	68.8 ± 13.2	80.8 ± 11.7*†		81.9 ± 12.0*	
Body height (m)	1.70 ± 0.10	1.72 ± 0.08		1.72 ± 0.08	
BMI (kgm^-2^)	23.7 ± 2.8	27.3 ± 3.6*†		27.7 ± 3.7*	

Kinetics		Affected	Non-affected	Affected	Non-affected
vGRF (Nkg^−1^)	4.95 ± 0.37	4.70 ± 0.52^#^	5.20 ± 0.52	4.91 ± 0.42	4.98 ± 0.44
HAM (Nmkg^−1^)	0.11 ± 0.08	0.10 ± 0.10^#^	0.16 ± 0.13	0.15 ± 0.11	0.14 ± 0.10
KAM (Nmkg^−1^)	0.01 ± 0.05	−0.01 ± 0.05^#^	0.02 ± 0.09	0.03 ± 0.06	0.02 ± 0.07

Kinematics		Affected	Non-affected	Affected	Non-affected
Lateral trunk displacement (°)	−0.4 ± 2.1	−0.4 ± 2.3		−0.1 ± 2.0	
Pelvic obliquity (°)	0.03 ± 1.0	−0.2 ± 2.3†		0.6 ± 1.9	
Hip abduction/adduction (°)	1.2 ± 2.0	2.0 ± 3.7	2.3 ± 4.4†	2.3 ± 3.0	1.2 ± 4.0
Knee flexion/extension (°)	2.0 (−1.8–4.3)	4.5 (0.9–8.0)†	0.6 (−2.4–5.8)†	1.6 (−1.2–3.9)	−0.3 (−3.2–3.1)
Ankle plantar/dorsiflexion (°)	5.9 (4.4–8.3)	5.1 (1.9–8.1)	5.3 (3.3–6.9)	5.1 (1.9–7.0)	5.1 (2.2–7.5)
Foot progression angle (°)	−8.4 (−11.3–−7.0)	−10.0 (−14.3–−7.9)†	−9.4 (−14.7–−5.4)	−8.8 (−11.5–−6.0)	−8.4 (−11.1–−6.1)

Values are mean values ± standard deviation, or median and interquartile range in parenthesis. The comparison between patients and healthy controls was tested with a chi-squared test (sex), Mann-Whitney test (age) and independent-sample *t*-tests (weight, height and body mass index (BMI)). Kinetic and kinematic differences between limbs were tested with dependent *t*-tests or Wilcoxon signed rank tests.

* Significant difference between patients and healthy controls.

# Significant difference between affected and non-affected limb.

† Significant difference between pre and postoperative values.

Negative values indicate ipsilateral thorax displacement, ipsilateral pelvic drop, hip abduction, knee extension, plantarflexion and external foot progression angle.

Abbreviations: OA, osteoarthritis; vGRF, vertical Ground Reaction Force; HAM, external hip adduction moment; KAM, external knee adduction moment.

Seventeen participants with a similar age distribution were included as a HC collective for comparison (Table 1). Control participants were included if they had no history of orthopedic surgeries or chronic and neuromuscular disease. All patients and HCs provided written informed consent prior to participation in the original studies. The protocol was approved by the Medical Ethics Committee of the Department of Medicine, Goethe University Frankfurt (reference number 122/14 and 497/15).

### 2.2 Bipedal standing

An 8-camera Vicon System operating at 200 Hz (8MX T10 cameras, VICON Motion Systems, Oxford, United Kingdom) collected kinematic data, synchronously with the two force plates (Advanced Mechanical Technology, Inc., Watertown, MA, United States). Reflective markers (14 mm) were placed on anatomical landmarks according to the standardized Plug-in-Gait marker set (Kadaba et al., 1990): pelvis (anterior and posterior superior iliac spines), upper leg (lateral thigh and lateral femoral condyle), lower leg (lateral shank and lateral malleolus), foot (heel and toe), shoulder (acromion) and thorax (sternum and spine). To improve the reliability and accuracy of the gait data in the frontal plane, additional markers were placed on the medial malleolus, medial femoral condyle and greater trochanter (Stief et al., 2013). The hip joint center was determined using a geometrical prediction method by Harrington (Harrington et al., 2007).

For the measurement, all participants were instructed to stand comfortably (barefoot) on their two legs for 10 s, with each foot resting on one of two force plates and arms at their sides. To achieve a natural balanced posture, no further instructions were given except when the thigh and pelvic markers were not visible, participants were asked to abduct their arms slightly.

Kinematic and kinetic data were exported to MATLAB for further analysis (version R2022a, The Mathworks Inc., Natick, MA, United States). The following kinematic outcome variables, based on the characteristic gait strategies (Schmidt et al., 2017), were extracted (Baker et al., 2018): mean LTD, mean pelvic obliquity, mean hip adduction, mean knee flexion/extension angle, mean ankle plantar/dorsiflexion angle and mean FPA. Here, negative LTDs refer to lateral displacements of the trunk with respect to the corresponding limb. Pelvic obliquity was negative when the pelvis dropped with respect to the corresponding limb. Adduction of the hip in the frontal plane was defined as a positive angle. Positive values for the knee indicated flexed knees, while negative values for the ankle indicated a plantar flexed ankle joint. FPA was defined as the angle of the long axis of the foot segment relative to the global coordinate system. Negative FPAs here indicate externally rotated feet. External joint moments were calculated from the force plate data and the mathematically derived joint centers by inverse dynamics analysis (Davis et al., 1991). Mean vertical ground reaction forces (vGRFs) of each limb and mean joint moments in the frontal plane for the hip and the knee joint (HAMs, KAMs) were normalized by body mass. Stance width was calculated as the distance between the ankle joint centers.

### 2.3 Symmetry angle

To quantify inter-limb symmetry with respect to vGRFs, HAMs, and KAMs, the symmetry angle (SA) was calculated using the following equation from Zifchock et al. (2008).

$$SA=\left|\frac{\left(45{}^{\circ}-\arctan\left(X_{L}/X_{R}\right)\right)}{90{}^{\circ}}\right|\times 100\%$$

Where XL and XR represent left/affected and right/non-affected limb values, respectively. SA values of 0% indicate perfect symmetry. SA values of 100% indicate two values that are opposite but equal in magnitude. The direction of asymmetry (indicated by a positive or negative value) was ignored and absolute values were used (Zifchock et al., 2008).

### 2.4 Statistical analyses

Statistical analyses were performed using SPSS Statistics (IBM SPSS Statistics for Windows, version 29, IBM Corp., Armonk, NY, United States). Shapiro-Wilk tests and visual inspection of Q-Q plots were used to check for normal distribution. Normally distributed parameters were compared with respect to differences between limbs (dependent *t*-test), groups (independent *t*-tests), and over time (dependent *t*-test). When data were not normally distributed, parameters were compared using Wilcoxon signed rank tests (differences between limbs and over time) and Mann-Whitney U-Tests (differences between groups). A chi-squared test was used to compare the sex distribution between groups.

The left and right sides of the HCs were randomized to a single-leg HC group for comparison with patients. Because significant HAM and KAM changes were expected in patients with unilateral hip OA, regression analysis was performed to determine predictor variables that best explained these changes. Pearson correlation coefficients were calculated to determine significant correlations between kinematic and kinetic predictor variables and HAMs and KAMs, respectively (Bortz, 1999). A stepwise multiple regression analysis was then performed if two or more parameters significantly correlated with the frontal external hip and knee joint moments. Secondary, a forward multiple regression analysis was performed using only the known compensatory strategy parameters (LTD, FPA and stance width). The level of significance was *α* = 0.05.

## 3 Results

Participant demographics are listed in Table 1. Patients were measured preoperatively and at a mean follow-up of 12.6 ± 2.5 months after surgery. The patients had significantly higher body mass and BMI compared to HCs, at preoperatory measure (80.8 vs. 68.8 kg, *p* < 0.001; 27.3 vs. 23.7 kgm^−2^; *p* < 0.001, respectively) and during follow-up (81.9 vs. 68.8 kg, *p* < 0.001; 27.7 vs. 23.7 kgm^−2^; *p* < 0.001, respectively). No differences were observed in age (preoperatory 63.0 vs. 56.0 years/postoperatory 64.0 vs. 56.0 years, *p* > 0.237), height (1.72 vs. 1.70 m, *p* > 0.379) or sex distribution (24 males and 19 females vs. 8 males and 9 females, *p* = 0.540) between the patients and HCs (Table 1). HCs stood with a stance width of 18.3 ± 4.3 cm, whereas patients stood with a stance width of 20.5 ± 4.3 cm before surgery and 20.1 ± 4.3 cm after surgery. The differences between HCs and patients were not significant (*p* > 0.074). Age (*p* < 0.001), body mass (*p* = 0.002) and BMI (*p* = 0.003) were significantly higher when postoperative anthropometrics were compared with preoperative values.

### 3.1 Kinetics and symmetry

Preoperatively, the non-affected limb carried more body weight than the affected limb in patients with unilateral hip OA (*p* = 0.003), as expressed by the vGRF (Table 1). In addition, greater HAMs (*p* = 0.007) and KAMs (*p* = 0.018) were found for the non-affected limb than for the affected limb. Postoperatively, no differences were found between the affected and non-affected sides (Table 1). Furthermore, no differences were found between HCs and patients pre- or postoperatively.

**FIGURE 1:**
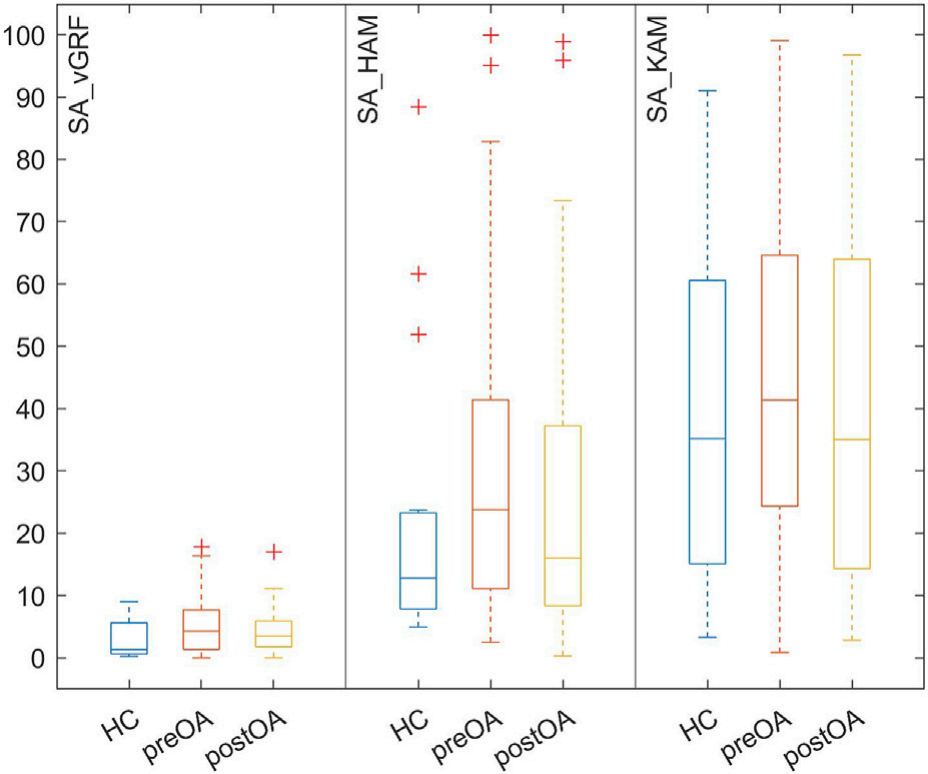
Box-and-whisker-plots of the absolute angles of symmetry (SA) of the vertical ground reaction force (vGRF) and external frontal hip (HAM) and knee (KAM) joint moments in healthy controls (HCs) and patients with hip osteoarthritis both preoperatively (preOA) and postoperatively (postOA).

During bipedal standing, no significantly increased symmetry angles (vGRF, HAM and KAM) were observed in the patients with unilateral hip OA compared with the HCs (Figure 1). Preoperatively, the symmetry angles were all higher for the patients and postoperatively the values all became smaller, but the differences between the patients and HCs never reached significance.

### 3.2 Kinematics

When considering the kinematics of patients with hip OA while standing on two legs (Table 1), it was found that the affected limb showed no difference from the non-affected limb both preoperatively and postoperatively. No differences were observed between HCs and patients, either preoperatively or postoperatively. Small significant changes were seen in the preoperative to postoperative comparison. The affected foot was less externally rotated, and the knees were less flexed or even extended after THR. In addition, the pelvis was no longer dropped to the affected side; postoperatively the pelvis dropped to the non-affected side with a corresponding change in hip adduction angle on the non-affected side.

### 3.3 Correlations and regression

All significant correlations to joint loading parameters (HAM and KAM) are shown in Table 2. Preoperatively, hip adduction angle and vGRF were significantly correlated with HAM in both the affected and non-affected limb. A significant correlation with stance width was also found for the affected limb. A significant correlation was found between KAM and stance width in the affected limb, and between KAM and vGRF in the non-affected limb.

**TABLE 2:** Results from the correlation analyses.

Dependent variable preoperative	Covariate	*r*	*p*-value
HAM affected leg	hip adduction	0.586	<0.001
	vGRF	0.456	0.002
	stance width	−0.443	0.003
HAM non-affected leg	hip adduction	0.635	<0.001
	vGRF	0.506	<0.001
KAM affected leg	stance width	−0.576	<0.001
KAM non-affected leg	vGRF	0.539	<0.001

Dependent variable postoperative	Covariate	*r*	*p*-value
HAM affected leg	hip adduction	0.588	<0.001
	vGRF	0.369	0.015
	stance width	−0.545	<0.001
	ankle flexion	0.353	0.020
HAM non-affected leg	hip adduction	0.642	<0.001
	stance width	−0.435	0.004
KAM affected leg	knee flexion	−0.353	0.020
	stance width	−0.409	0.006
KAM non-affected leg	knee flexion	−0.366	0.016

Abbreviations: HAM, external hip adduction moment; KAM, external knee adduction moment; vGRF, vertical Ground Reaction Force.

Postoperatively, the same significant correlations with HAM were found for the affected limb (hip adduction angle, vGRF and stance width). There was also a significant correlation with the ankle plantarflexion angle. For the non-affected limb, significant correlations with HAM were found for hip adduction angle and stance width. KAM significantly correlated with knee flexion for both the affected and the non-affected limb. A significant correlation with stance width was also found for the affected leg.

Preoperative regression analysis revealed that changes in hip adduction angle and vGRFs explained 42% of HAM changes (*R* ^2^ = 0.418; *F* = 14.360; *p* < 0.001) for the affected limb. Stance width did not significantly improve the outcome using this model (∆*R* ^2^ = 0.037, *∆F* = 2.673, *p* = 0.110). In the non-affected limb changes in hip adduction angle and vGRFs explained 61% of HAM changes (*R* ^2^ = 0.612; *F* = 31.569; *p* < 0.001).

Postoperatively, the regression analysis yielded a model in which hip adduction angle and vGRF explained 42% of HAM alterations (*R* ^2^ = 0.417; *F* = 14.310; *p* < 0.001). Adding stance width (likely due to relatively high correlation to hip adduction) and ankle flexion to the model did not result in a significant increase in the percentage of variance in HAM predicted by the model. For the non-affected limb, hip adduction angle explained 41% of the changes in HAM (*R* ^2^ = 0.413; *F* = 28.808; *p* < 0.001), and adding stance width did not significantly increase the outcome using this model (∆*R* ^2^ = 0.014, *∆F* = 1.004, *p* = 0.322). For the affected leg only 16% of the changes in KAM were explained by the changes in stance width (*R* ^2^ = 0.167; *F* = 8.245; *p* = 0.006), the inclusion of the knee flexion angle did not significantly increase the percentage of variance in KAM predicted by the model (∆*R* ^2^ = 0.062, *∆F* = 3.198, *p* = 0081).

Multiple regression analysis with only known parameters of the compensatory strategies showed that only stance width explained part of the variation of the external joint moments in the frontal plane (see Table 2). The other parameters (LTD and FPA) did not significantly improve the outcome using any of the models.

## 4 Discussion

Altered joint moments in individuals with unilateral hip OA may contribute to the development of OA in adjacent joints (Jungmann et al., 2015). To identify possible abnormal joint moments in the knee and hip joint during standing, this study quantified weight distribution and lower limb joint moments in individuals with unilateral hip OA during bipedal standing before (preoperatively) and 1 year after THR (postoperatively). The results of the current study suggest that weight distribution favoring the affected limb and few kinematic adjustments in the affected limb enable patients with unilateral hip OA to stand comfortably while standing on two legs before THR. At a mean follow-up of 12.6 months, asymmetries in joint loading were no longer present.

### 4.1 Kinetics and symmetry

**FIGURE 2:**
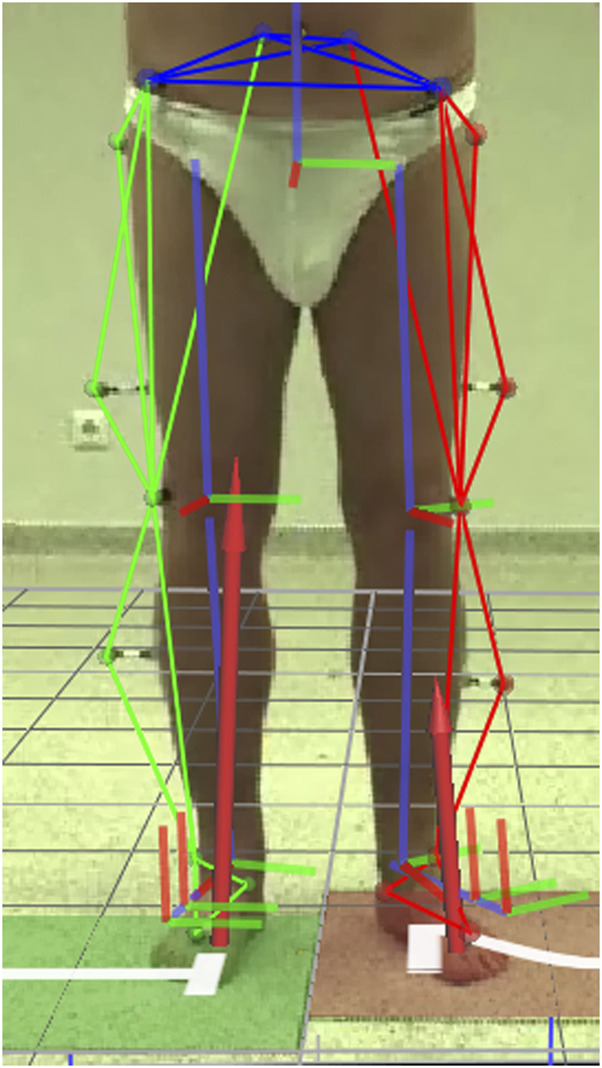
Patient with unilateral hip osteoarthritis (preoperatively) in a standing position with a frequently observed extended external foot rotation on the affected (left) side. Kinematic segments and ground reaction force are superimposed on the image.

To avoid pain caused by the affected hip, individuals with unilateral hip OA appear to adopt an altered bipedal standing position (Figure 2), resulting in a redistribution of lower limb joint moments. As previously noted, the non-affected limb carried more body weight than the affected limb in patients with unilateral hip OA (Talis et al., 2008; Miura et al., 2018). The non-affected limb experienced 38% greater HAMs than the affected limb, indicating a pathological loading of the hip joint. KAM tended to be a valgus moment in the affected limb, which denoted that the vertical GRF vector was slightly lateral from the estimated knee joint center rather than slightly medial, which has been shown to be physiological during walking (Cerny, 1984). During walking, patients with unilateral hip OA exhibited pathological peak external KAM and HAM in the non-affected limb (Hurwitz et al., 1997; Shakoor et al., 2011; Schmidt et al., 2017; Stief et al., 2018). Therefore, it has been suggested that the non-affected limb is at higher risk for developing OA in these joints (Hurwitz et al., 2001). Comparable results have been obtained during sit-to-stand tasks (Eitzen et al., 2014; Abujaber et al., 2015) and stair climbing (Queen et al., 2015).

Miura et al. (2018) showed that the load distribution of the operated leg was restored as early as 1 month after surgery. In the present study it was found that the load distribution and corresponding joint moments had no asymmetries between limbs at a follow-up of 12.6 months on average. Postoperative joint moments were significantly different from preoperative values [i.e., HAM appeared to be 33% higher in the affected leg after surgery (0.15 vs. 0.10 Nmkg^−1^)]. No differences were observed compared with HCs. Studies also found increased HAM during walking compared to HCs (Stief et al., 2018), although in general, peak HAM values were usually lower but not significantly reduced after THR compared to HCs (Ewen et al., 2012). The increase in HAM may be due to the absence of pain and the resultant increase in vGRF after THR. However, whether other factors such as an altered moment arm due to repositioning of the hip joint center during THR surgery play a role requires further investigation.

### 4.2 Kinematics and correlations

In this study, it was hypothesized that patients with unilateral hip OA adopt a standing position that is significantly different from HCs to reduce the moments in the affected hip joint. However, HAMs in the affected limb were not reduced compared to normal, which partly explains the lack of significant kinematic changes compared with HCs. For example, during walking, patients with unilateral hip OA significantly bend their trunk toward the affected side (Bennett et al., 2007; Reininga et al., 2012). Consequently, HAM in the affected limb was significantly reduced during gait (Hurwitz et al., 1997; Foucher et al., 2007; Shakoor et al., 2011; Foucher and Wimmer, 2012). When standing on two legs, patients with hip OA showed minimal LTD to the affected side, which was not significantly different from HCs. Bending of the trunk towards the affected limb during bipedal standing may be detrimental in terms of postural stability or even non-feasible while at the same time the non-affected limb carries proportionally more body weight. Preoperatively, the foot of the affected limb was significantly more externally rotated during bipedal stance (Figure 2; Table 1) while the knee was more flexed (Table 1) when compared with the postoperative standing position, but not when compared with HCs. Although these kinematic changes during standing did not relate to changes in HAM, they appear to be adopted by patients with unilateral hip OA to unload the affected limb in terms of body weight distribution. Increased FPAs and knee flexion changes are typical of patients with unilateral hip OA during walking (Bennett et al., 2007; Müller et al., 2012; Schmitt et al., 2015). Previous studies on HCs have shown that large changes in LTD (Mündermann et al., 2008) and FPA (Andrews et al., 1996) are required to significantly reduce KAMs during walking. Hip adduction and plantar/dorsiflexion angle of the ankle are known control variables of sway in the medio-lateral and anterior-posterior directions, respectively (Weaver et al., 2017). Therefore, it makes sense that these variables are predictors of the hip joint moments during stance. In the present study, no kinematic differences were found between the affected and non-affected limb. The relatively small kinematic changes observed in the affected limb may be the reason why kinematics did not explain the KAM alterations preoperatively. It appears that vGRF, hip adduction and knee flexion angle explain most of the variance in HAM and KAM. However, stance width also showed correlations to HAM and KAM and since increased stance width influences hip adduction angle, stance width indirectly contributes to explain the variance. When only compensation parameters were considered, stance width was the only parameter that mattered: stance width explained 18.9%–29.7% of the variance of HAM and 16.7%–33.2% of the variance of KAM (Table 2). From walking it is known that increasing stride width reduces knee and hip joint moments in the frontal plane (Stief et al., 2021), so increasing stance width could be a simple adaptation with a reducing effect on knee and hip joint moments in the frontal plane during standing.

### 4.3 Limitations

This study should be considered in the context of its limitations. Only variables that have been shown to correlate with external KAM and HAM during walking were examined. However, it is likely that patients with unilateral hip OA modify additional kinematic and kinetic parameters to stand comfortably on two legs. In addition, factors such as pain, leg length discrepancy, and pelvic or spinopelvic malalignment have been suggested to contribute toasymmetric loading (Miura et al., 2018) and were not investigated in the current study.

KAM was found to be very small because the GRF was centered on the center of rotation of the knee joint, in contrast to gait where the GRF is directed medially from the center of rotation of the knee joint (Cerny, 1984). Therefore, KAM and consequently the SA for KAM may not be the best parameters for assessing knee loading during bipedal standing. Although knee and hip joint moments in the frontal plane during dynamic activities have been widely used as an indicator of knee joint loading and to characterize intrinsic compressive loading, this parameter itself may not be sufficient to predict the mechanical properties of the cartilage, especially during standing. Therefore, future studies are needed to analyze possible relationships between knee and hip joint loading parameters and compressive properties of joint cartilage, i.e., a combination of musculoskeletal and finite element models (Harlaar et al., 2022).

## 5 Conclusion

In summary, our elderly patients with unilateral hip OA adopted a standing position preoperatively in which foot progression (more externally rotated) and knee flexion (more flexed) were adapted in the affected limb compared to the postoperative standing position. However, these kinematic adaptations did not explain the lower HAM and KAM in the affected limb compared with the non-affected limb. The non-affected limb experienced 38% greater HAMs than the affected limb, indicating pathological loading of the hip joint during normal standing. Postoperatively, limb load distribution and corresponding joint loads normalized, comparable to findings during walking. The altered preoperative joint moments were mainly caused by a disproportionate weight distribution, expressed by the vGRF, in favor of the affected limb. Of the compensation strategy parameters, stance width showed moderate to high correlations with HAM and KAM and also explained up to 33% of the variance. Therefore, in patients with hip OA, interdisciplinary preventive therapy concepts in which stance width could play a role should be considered to improve the loading situation.

## Data Availability

The raw data supporting the conclusion of this article will be made available by the authors, without undue reservation.
